# Kidney function and specific mortality in 60-80 years old post-myocardial infarction patients: A 10-year follow-up study

**DOI:** 10.1371/journal.pone.0171868

**Published:** 2017-02-09

**Authors:** Ellen K. Hoogeveen, Johanna M. Geleijnse, Erik J. Giltay, Sabita S. Soedamah-Muthu, Janette de Goede, Linda M. Oude Griep, Theo Stijnen, Daan Kromhout

**Affiliations:** 1 Department of Nephrology, Jeroen Bosch Hospital, Den Bosch, the Netherlands; 2 Department of Epidemiology, Leiden University Medical Center, Leiden, the Netherlands; 3 Department of Nephrology, Leiden University Medical Center, Leiden, the Netherlands; 4 Division of Human Nutrition, Wageningen University, Wageningen, the Netherlands; 5 Department of Psychiatry, Leiden University Medical Center, Leiden, the Netherlands; 6 Department of Medical Statistics and Bioinformatics, Leiden University Medical Center, Leiden, the Netherlands; The University of Tokyo, JAPAN

## Abstract

Chronic kidney disease (CKD) is highly prevalent among older post-myocardial infarction (MI) patients. It is not known whether CKD is an independent risk factor for mortality in older post-MI patients with optimal cardiovascular drug-treatment. Therefore, we studied the relation between kidney function and all-cause and specific mortality among older post-MI patients, without severe heart failure, who are treated with state-of-the-art pharmacotherapy. From 2002–2006, 4,561 Dutch post-MI patients were enrolled and followed until death or January 2012. We estimated Glomerular Filtration Rate (eGFR) with cystatin C (cysC) and creatinine (cr) using the CKD-EPI equations and analyzed the relation with any and major causes of death using Cox models and restricted cubic splines. Mean (SD) for age was 69 years (5.6), 79% were men, 17% smoked, 21% had diabetes, 90% used antihypertensive drugs, 98% used antithrombotic drugs and 85% used statins. Patients were divided into four categories of baseline eGFR_cysC_: ≥90 (33%; reference), 60–89 (47%), 30–59 (18%), and <30 (2%) ml/min/1.73m^2^. Median follow-up was 6.4 years. During follow-up, 873 (19%) patients died: 370 (42%) from cardiovascular causes, 309 (35%) from cancer, and 194 (22%) from other causes. After adjustment for age, sex and classic cardiovascular risk factor, hazard ratios (95%-confidence intervals) for any death according to the four eGFR_cysC_ categories were: 1 (reference), 1.4 (1.1–1.7), 2.9 (2.3–3.6) and 4.4 (3.0–6.4). The hazard ratios of all-cause and cause-specific mortality increased linearly below kidney functions of 80 ml/min/1.73 m^2^. Weaker results were obtained for eGFR_cr_. To conclude, we found in optimal cardiovascular drug-treated post-MI patients an inverse graded relation between kidney function and mortality for both cardiovascular as well as non-cardiovascular causes. Risk of mortality increased linearly below kidney function of about 80 ml/min/1.73 m^2^.

## Introduction

Chronic kidney disease (CKD), defined by an estimated glomerular filtration rate (eGFR) <60 ml/min/1.73m^2^, is an increasing global public health problem that affects about 25% of people at age 65–74 years and >50% at age 75 years or older.[1,2] Kidney function decreases with age by approximately 10 ml/min per decade after age 40 years, even in the absence of important risk factors for CKD such as smoking, diabetes, hypertension and proteinuria.[3] The growth in number of older people (age >65 years) and the increased incidence of diabetes, hypertension and obesity, contributes to the increased prevalence of CKD.[4,5] In addition, previous studies showed that the rate of kidney function decline is at least twice as high in cardiac patients compared with the general population.[6,7] Therefore, post-myocardial infarction patients have an increased risk of CKD.

CKD is an established risk factor for all-cause and cardiovascular mortality in younger patients.[8] A recent meta-analysis showed that a 30% lower estimated glomerular filtration rate (eGFR) was associated with an approximately 30% greater risk of death in patients without or with a history of vascular disease.[9] Unfortunately, this meta-analysis did not report specific risks per age-category. Another meta-analysis, including cohorts of the general population, high-risk and CKD patient populations, showed a greater mortality risk below an eGFR of 60–75 ml/min/m^2^ in every age category.[10] In contrast, other studies showed that in older patients relative risk for death increased below a much lower threshold of kidney function of about 45–50 ml/min/1.73m^2^.[3,11] At older ages mortality risks were smaller on the relative scale, but greater on the absolute scale.[12]

Clinical guidelines recommend that patients with CKD should be treated for cardiovascular risk to ameliorate progression of CKD and improve patient outcome.[8] However, the strong relation of age with incident CKD is a part of a long-standing debate about the question whether kidney function decline is "normal aging" or a pathologic process.[12,13] Given the controversy about whether CKD is an independent risk factor of mortality in older patients, we studied the shape and strength of the relation between kidney function and all-cause, as well as specific causes of mortality, in a cohort of state-of-the-art drug-treated patients aged 60–80 years with a verified history of myocardial infarction.[14] The cohort was followed up to 10 years.

## Methods

### Study population

This prospective cohort study is a follow-up of the Alpha Omega Trial, to explore the associations of estimated kidney function on the risk of all-cause and specific causes of death. The Alpha Omega Trial is a randomized controlled trial of omega-3 (n-3) fatty acids supplementation in 4,837 patients with a verified history of myocardial infarction (MI), no severe heart failure, as described in detail elsewhere.[14,15] Presence of cancer with <1 year of life expectancy was an exclusion criterion for the Alpha Omega Trial.[15] Briefly, in the Alpha Omega Trial we randomly assigned patients to four trial margarines with marine n-3 fatty acids eicosapentaenoic acid (EPA) and docosahexaenoic acid (DHA), plant-derived alpha-linolenic acid (ALA), or placebo. The patients received an additional targeted amount of either 400 mg/day EPA and DHA, or 2 g/day ALA, or EPA-DHA plus ALA, or placebo for 40 months. In January 2002, the Alpha Omega Trial was approved by a central medical ethics committee (Haga Hospital, Leyenburg in the Hague, the Netherlands) and by the ethics committee at each participating hospital*. Patients were enrolled from April 2002 through December 2006. The trial started in 2002 and was closed in 2009, but the mortality follow-up was continued until January 2012. The Alpha Omega Trial is a non-drug trial. When the first patients were enrolled in 2002, trial registration of dietary intervention studies was not common practise. The first international policy on trial registration was introduced by the International Committee of Medical Journal Editors in October 2004.[16] In August 2005, we registered our trial (ClinicalTrials.gov number: NCT00127452). For the present observational analysis data were available of 4,561 free-living cardiac patients aged 60–80 years of whom both serum cystatin C (cysC) and creatinine (cr) were determined at baseline. This study was conducted in accordance with the Helsinki Declaration. Written informed consent was obtained from all patients.

### Kidney function assessment

Standardized blood handling procedures for the Alpha Omega Trial were described in detail elsewhere.[17] Briefly, non-fasting blood samples were obtained at the subjects' home or at the hospital. Tubes were packaged in sealed envelopes and sent via standard postal service to a central laboratory. At baseline serum cystatin C (cysC) as well as creatinine (cr) were measured from stored blood samples in a central laboratory as described elsewhere.[7] In short, serum cysC was measured by means of a particle-enhanced immunonephelometric assay (N Latex cystatin C; Dimension Vista 1500 analyzer, Siemens) traceable to the International Federation of Clinical Chemistry Working Group for Standardization of Serum Cystatin C.[18] We used calibrators and assays of the same lot-code, which was stable (no downward drift). CysC was calibrated directly using the standard supplied by the manufacturer. The analytical measurement range of cysC was: 0.23 to 8.00 mg/L. Intra- and inter-assay variation for low cysC (mean = 1.00 mg/L) were 1.3% and 4.2%, respectively, and for high cysC (mean = 1.75 mg/L) they were 2.9% and 2.8%, respectively. Serum creatinine (cr) was measured by the modified kinetic Jaffé method (Dimension Vista 1500 analyzer, Siemens). We calibrated directly to the standard supplied by the manufacturer from the National Institute of Standards and Technology-Standard Reference material, and a post-calibration correction factor was applied.[19]

### Risk factors

Patients were interviewed and physically examined by trained research nurses at home or in the hospital at baseline. Information on demographic variables, lifestyle habits, current health status and medical history were collected by self-administered questionnaires as previously described.[15] History of symptomatic heart failure was self-reported and collected by a self-administered questionnaire as follows: "Did a physician ever establish the diagnosis heart failure (decompensatio cordis): yes, no, or do not know". Medication was coded according to the Anatomical Therapeutic Chemical Classification System (ATC). At baseline, anthropometric measures were assessed and blood pressure was measured. Diabetes mellitus was considered present in case of a self-reported physician diagnosis, use of antidiabetic drugs and/or elevated blood glucose. Lipid and glucose levels were determined, as described elsewhere.[14] High-sensitivity C-reactive protein (hsCRP) levels were measured in stored serum samples, as previously described.[20]

### Outcome

Patients were followed until death or censored at January 1, 2012. The vital status of the participants was monitored via a computerized link with municipal registries. No one was lost to follow-up. Information on the primary causes of death was obtained from the Dutch National Mortality Registry (Statistics Netherlands [CBS]) from May 2002 through January 2012. Causes of death were coded according to the International Classification of Diseases, 10^th^ revision. Cardiovascular mortality included diseases of the circulatory system (I00-I99) and sudden death undefined (R96), mortality due to cancer included all malignant neoplasms (C00-C97), and all other causes of death were called "non-cardiovascular-non-cancer".

## Statistical analysis

Variables are presented as mean (standard deviation [SD]), median (interquartile range [IQR]), or number (proportion) where appropriate. We estimated GFR with the cystatin C-based Chronic Kidney Disease Epidemiology Collaboration (CKD-EPI) equation from 2012, recommended by the KDIGO 2012 guideline as the confirmatory test, taking into account age, sex and race.[21,22] Results for the creatinine-based CKD-EPI equation from 2009 and combined cystatin C-creatinine based eGFR calculated with the CKD-EPI equation from 2012, are presented in the Supporting Information. Patients were divided into four categories on the basis of their baseline cysC-based kidney function: eGFR: ≥90 (reference), 60–89, 30–59 and <30 ml/min/1.73m^2^. First, absolute mortality rates were calculated within each eGFR category. Second, we conducted Cox proportional hazards analysis, obtaining hazard ratios (HR) and 95% Confidence Intervals (CI) estimating the strengths of associations for various eGFR categories with mortality using eGFR ≥90 ml/min/1.73m^2^ as reference. The proportional hazards assumption was fulfilled in all models according to the graphical approach and Schoenfeld residuals. Analyses were adjusted for the n-3 fatty acid treatment groups of the Alpha Omega Trial (using 3 dummies: placebo vs three active treatments) and for the following potential confounders: age, sex, diabetes, current smoking, serum total/HDLcholesterol ratio, use of statins and of anti-hypertensive medication, and systolic and diastolic blood pressure (model 1). To evaluate a possible modifying role of risk factors, we repeated the previous analyses in strata of sex, diabetes, current smoking, alcohol use and physical activity. In a separate analysis we adjusted additionally for hsCRP (model 2), since the level of CRP is weakly associated with cysC.[23] Third, to study the continuous relation between kidney function and mortality in a very flexible way we employed four-knot restricted cubic splines with 95%-CIs. According to general guidelines (e.g. Harrell [24]), the knots were chosen at the 5th, 35th, 65th and 95th percentile of the kidney function distribution, corresponding to cystatin C-based eGFR of 42, 73, 89, and 108 ml/min/1.73m^2^. An eGFR of 120 ml/min/1.73m^2^ was taken as the reference point (HR = 1.00). We adjusted for the previous mentioned potential confounders (model 1). Age was included linearly in the model, since addition of a quadratic term did not improve the model. All analyses were done using SPSS 24.0 (SPSS, Inc. Chicago, IL).

## Results

Baseline characteristics of all patients according to four categories of cysC-based eGFR at baseline are presented in Table 1. The mean (SD) age of the study cohort was 69 (6) years, 79% were men, 99.5% were white, 21% had diabetes, 24% were obese, and 17% smoked. The median (IQR) interval after MI was 3.7 years (1.7–6.3) at baseline. The mean (SD) systolic and diastolic blood pressure (BP) was 142 (22) mmHg and 80 (11) mmHg, respectively. Of all patients, 90% used blood pressure lowering drugs, of whom 56% used renin-angiotensin blockers, 98% used antithrombotic drugs and 85% used statins. Mean (SD) serum cysC was 1.01 (0.30) mg/L and serum creatinine was 1.07 (0.40) mg/dl, and mean (SD) eGFR_cysC_ was 78.8 (20.6) and eGFR_cr_ was 82.7 (21.3) ml/min/1.73m^2^. At baseline mean (SD) kidney function did not differ among the four treatment groups (placebo, ALA, EPA-DHA and EPA-DHA plus ALA), being for eGFR_CysC_ 79.3 (20.4), 79.3 (20.7), 78.7 (20.9) and 77.8 (20.4) ml/min/1.73m^2^ (P = 0.3), respectively, and for eGFR_cr_ 83.0 (21.1), 83.8 (21.4), 81.9 (21.2) and 82.2 (21.5) ml/min/1.73m^2^ (P = 0.1), respectively. The correlation of log-transformed hsCRP with eGFR_cysC_ was -0.29 and with eGFR_cr_ -0.10 (both P<0.001).

**Table 1:** Baseline characteristics of the cohort of 4561 post-myocardial infarction patients according to four categories of cystatin C-based estimated glomerular filtration rate (eGFR).

Cystatin C-based eGFR, ml/min/1.73m^2^	≥90	60–89	30–59	<30
	(n = 1517, 33.3%)	(n = 2162, 47.4%)	(n = 809, 17.7%)	(n = 73, 1.6%)
Age, y	66.1 ± 4.5	69.5 ± 5.3	73.0 ± 5.0	73.4 ± 4.9
Men, No. (%)	1335 (88.0)	1665 (77.0)	570 (70.5)	50 (68.5)
Ethnicity, white No. (%)	1504 (99.1)	2156 (99.7)	806 (99.6)	72 (98.6)
Higher education^a^, No (%)	222/1514 (15)	258/2143 (12)	83/802 (10)	7/72 (10)
Physical active^b^ (≥3 MET during >5d/wk), No. (%)	369/1510 (24)	470/2151 (22)	120/801 (15)	7/72 (10)
Current Smoker, No. (%)	220 (14.5)	373 (17.3)	147 (18.2)	17 (23.3)
Alcohol use ≥1 glass/week, No (%)	1263/1515 (83)	1578/2158 (73)	505/805 (63)	40/73 (55)
Time since MI, y	4.3 ± 3.0	4.3 ± 3.3	4.2 ± 3.3	4.5 ± 2.8
Self-reported history of heart failure, No. (%)				
Yes	332/1455 (23)	535/2042 (26)	312/781 (40)	36/69 (52)
No	870/1455(60)	1082/2042 (53)	307/781 (39)	27/69 (39)
Do not know	253/1455 (17)	425/2042 (21)	162/781 (21)	6/69 (9)
Self-reported history of stroke, No. (%)	66/1509 (4.4)	154/2144 (7.2)	92/796 (11.6)	12/73 (16.4)
Diabetes^c^, No. (%)	286 (18.9)	411 (19.0)	221 (27.3)	27 (37.0)
Antidiabetic drugs, No (%)	201 (13.2)	296 (13.7)	168 (20.8)	20 (27.4)
Body mass index^d^, kg/m^2^	27.4 ± 3.5	27.8 ± 3.7	28.0 ± 4.4	29.8 ± 6.3
≥30 kg/m^2^, No (%)	307 (20.3)	527 (24.4)	225 (27.9)	26 (35.6)
Systolic blood pressure, mmHg	142 ± 20	142 ± 22	142 ± 24	143 ± 27
Diastolic blood pressure, mmHg	82 ± 11	80 ± 11	77 ± 12	75 ± 13
Use of cardiovascular medication^e^, No. (%)				
Antithrombotic agents	1497 (99)	2110 (98)	772 (95)	71 (97)
Antiplatelet drugs	1378 (91%)	1813 (84%)	590 (73%)	47 (64%)
Blood pressure lowering drugs	1289 (85.0)	1960 (90.7)	760 (93.9)	72 (98.6)
ACE-inhibitor and/or Angiotensin blocker	752 (49.6)	1239 (57.3)	526 (65.0)	56 (76.7)
Beta-blockers	993 (65.5)	1525 (70.5)	554 (68.5)	60 (82.2)
Diuretics	134 (8.8)	503 (23.3)	426 (52.7)	47 (64.4)
Lipid modifying drugs	1355 (89)	1874 (87)	633 (78)	62 (85)
Glucose^f^, mg/dl	111 (37)	111 (37)	115 (42)	118 (44)
Cholesterol (total)^g^, mg/dl	182 ± 35	182 ± 38	185 ± 40	183 ± 39
LDL, mg/dl	100 ± 31	100 ± 33	101 ± 34	95 ± 35
HDL, mg/dl	50 ± 13	50 ± 13	48 ± 13	45 ± 12
Triglycerides^h^, mg/dl	138 (101 to 195)	146 (108 to 204)	159 (119 to 213)	172 (135 to 278)
High-sensitivity C-reactive protein, mg/L	1.2 (0.6 to 2.6)	1.8 (0.9 to 3.8)	3.1 (1.3 to 5.9)	3.5 (1.8 to 7.9)
Serum cystatin C, mg/L	0.79 ± 0.06	0.98 ± 0.08	1.36 ± 0.19	2.43 ± 0.64
Cystatin C-based^i^ eGFR ml/min/1.73m^2^	100.2 (94.9 to 105.4)	77.0 (68.5 to 83.1)	51.3 (43.2. to 56.9)	25.2 (20.8 to 27.7)
Serum creatinine^j^, mg/dl	0.89 ± 0.22	1.05 ± 0.28	1.35 ± 0.36	2.65 ± 1.00
Creatinine-based^k^ eGFR ml/min/1.73m^2^	96.4 (90.0 to 101.2)	85.7 (71.5 to 95.2)	62.1 (50.0 to 76.4)	29.3 (22.6 to 37.3)

Data are presented as median (interquartile range), mean (±SD) or number (percentage of the total).

^a.^ Defined as higher vocational education or university.

^b.^ Defined as ≥3 Metabolic Equivalent Tasks (MET) during >5d/wk.

^c.^ Diabetes was considered to be present if a patient reported having received the diagnosis from a physician, was taking antidiabetic drugs, or had an elevated plasma glucose level (≥126 mg/dl in the case of patients who had fasted more than 4 hours or ≥200 mg/dl in the case of nonfasting patients).

^d.^ Body mass index was calculated as weight in kilograms divided by height in meters squared.

^e.^ Antithrombotic agents ATC code B01. Antiplatelet drugs ATC code B01AC. Blood pressure lowering drugs ATC codes C02, C03, C07, C08 and C09. Lipid modifying drugs ATC code C10.

^f.^ To convert the values for glucose to mmol/L, multiply by 0.05551.

^g.^ To convert the values for cholesterol to mmol/L, multiply by 0·02586.

^h.^ To convert the values for triglycerides to mmol/L, multiply by 0.01129.

^i^. Cystatin C-based CKD-EPI equation 2012.[22]

^j.^ To convert the values for creatinine to micromoles per liter, multiply by 88.40.

^k.^ Creatinine-based CKD-EPI equation 2009.[22]

### All-cause mortality

**Fig 1:**
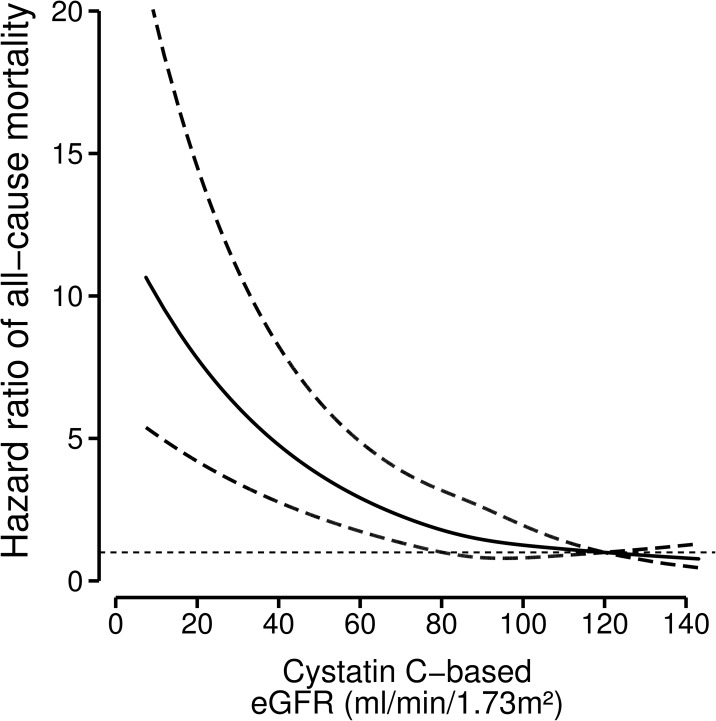
Relation between baseline kidney function and all-cause mortality with 95%-confidence intervals (dotted lines) among 4561 post-myocardial infarction patients. Hazard ratios for all-cause mortality depending on kidney function were modeled by separate restricted cubic splines for cystatin C-based kidney function in a Cox-regression model. The model was adjusted for n-3 fatty acids treatment, age, sex, diabetes, current smoking, ratio serum cholesterol-HDL, statin-use, use of anti-hypertensive medication, systolic and diastolic blood pressure. An eGFR of 120 ml/min/1.73m^2^ was taken as the reference point (hazard ratio 1).

We accrued 28,840 person-years of follow-up. During a median follow-up of 6.4 y (IQR: 5.3 to 7.6), 873 (19.1%) patients died. The absolute mortality rates (95%-CI) per 100 person-years (py) in post-MI patients for the four eGFR_cysC_ are presented in Table 2. The crude mortality rate in patients with eGFR_cysC_ of 60–89, 30–59, and <30 ml/min/1.73m^2^ compared with the reference was 1.8, 4.7 and 6.7 times higher, which corresponded to an excess rate of 1.2, 5.5 and 8.6 deaths/100 py. Using proportional hazards regression analysis we found after multivariable adjustment a graded inverse relation between kidney function and all-cause mortality (Table 2 and S1 and S2 Tables). Additional adjustment for self-reported stroke or BMI ≥30 kg/m^2^ did not attenuate the results. After stratification for treatment with or without fish oil (EPA-DHA) and multivariable adjustment we found no differences in risk for all-cause mortality among the four eGFR categories (P for interaction = 0.2). After stratification by age, diabetes, history of self-reported stroke, smoking, alcohol use, physical activity we found no evidence for effect modification. Fig 1 (S1 and S2 Figs) shows the continuous relation between kidney function and all-cause mortality rates (with 95%-CIs) expressed by the hazard ratio after multivariable adjustment. Risk of all-cause mortality increased significantly below an eGFR of approximately 80 ml/min/1.73m^2^.

**Table 2:** Mortality risks according to four categories of cystatin-C based estimated glomerular filtration rate (eGFR).

Cystatin C-based eGFR, ml/min/1.73m^2^	≥90	60–89	30–59	<30	P for Trend
**All-cause mortality**					
No patients	1517	2162	809	73	
Person-years (py)	10191.26	13804.35	4487.60	357.20	
No deaths	154	367	316	36	
AR per 100 py (95%-CI)	1.51 (1.29 to 1.77)	2.66 (2.40 to 2.94)	7.04 (6.33 to 7.83)	10.08 (7.37 to 13.64)	
Crude	1	1.79 (1.48 to 2.16)	4.93 (4.07 to 5.98)	7.30 (5.08 to 10.50)	<0.001
Age & sex adj.	1	1.46 (1.20 to 1.77)	3.31 (2.67 to 4.11)	4.80 (3.29 to 7.00)	<0.001
Model 1	1	1.37 (1.12 to 1.67)	2.85 (2.28 to 3.55)	4.38 (2.99 to 6.42)	<0.001
Model 2	1	1.33 (1.09 to 1.63)	2.74 (2.20 to 3.42)	4.15 (2.83 to 6.09)	<0.001
**Cardiovascular mortality**					
No deaths	57	150	145	18	
Crude	1	1.97 (1.45 to 2.67)	6.10 (4.49 to 8.29)	9.78 (6.75 to 16.62)	<0.001
Age & sex adj.	1	1.62 (1.18 to 2.22)	4.15 (2.95 to 5.83)	6.50 (3.74 to 11.30)	<0.001
Model 1	1	1.55 (1.13 to 2.14)	3.61 (2.55 to 5.10)	6.04 (3.44 to 10.58)	<0.001
Model 2	1	1.52 (1.11 to 2.10)	3.49 (2.47 to 4.95)	5.76 (3.28 to 10.11)	<0.001
**Cancer mortality**					
No deaths	71	142	91	5	
Crude	1	1.50 (1.13 to 2.00)	3.07 (2.25 to 4.19)	2.20 (0.89 to 5.45)	<0.001
Age & sex adj.	1	1.30 (0.97 to 1.76)	2.31 (1.63 to 3.28)	*	
Model 1	1	1.23 (0.91 to 1.66)	2.11 (1.48 to 3.01)	*	
Model 2	1	1.20 (0.89 to 1.62)	2.02 (1.41 to 2.88)	*	
**Non-cardiovascular/non-cancer mortality**					
No deaths	26	75	80	13	
Crude	1	2.17 (1.39 to 3.39)	7.49 (4.81 to 11.66)	15.90 (8.16 to 30.99)	<0.001
Age & sex adj.	1	1.56 (0.98 to 2.48)	4.02 (2.46 to 6.58)	8.41 (4.18 to 16.94)	<0.001
Model 1	1	1.38 (0.87 to 2.20)	3.11 (1.89 to 5.12)	6.72 (3.29 to 13.70)	<0.001
Model 2	1	1.35 (0.85 to 2.15)	3.00 (1.82 to 4.95)	6.38 (3.12 to 13.04)	<0.001

AR absolute risk, CI confidence interval, No number, py person years, Model 1: adjusted for the intervention with n-3 fatty acids, age, sex, diabetes, current smoking, ratio serum total cholesterol/HDL, statin-use, anti-hypertensive medication, systolic blood pressure, and diastolic blood pressure. Model 2: in addition to Model 1 additional adjustment for C-reactive protein. eGFR **≥**90 ml/min/1.73m^2^ was taken as the reference category.

*Due to the low number of events in the lowest category of eGFR further adjustment could not be performed.

### Specific causes of death

**Fig 2:**
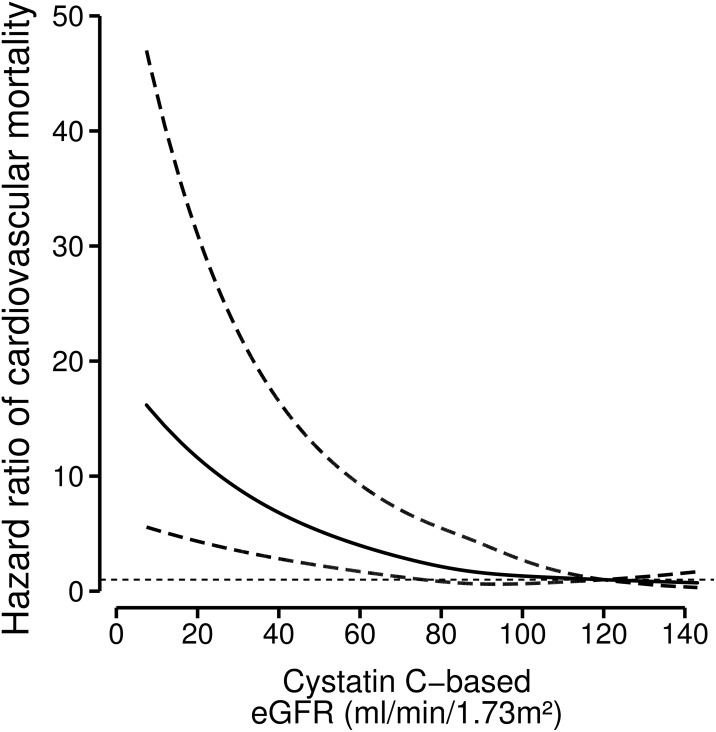
Relation between baseline kidney function and cardiovascular mortality with 95%-confidence intervals (dotted lines) among 4561 post-myocardial infarction patients. Hazard ratios for cardiovascular mortality depending on kidney function were modeled by separate restricted cubic splines for cystatin C based-kidney function in a Cox-regression model. The model was adjusted for n-3 fatty acids treatment, age, sex, diabetes, current smoking, ratio serum cholesterol-HDL, statin-use, use of anti-hypertensive medication, systolic and diastolic blood pressure. An eGFR of 120 ml/min/1.73m^2^ was taken as the reference point (hazard ratio 1).

Of all patients who died, 370 deaths (42%) were cardiovascular. We found a strong inverse graded relation between eGFR_cysC_ and cardiovascular death (Table 2). Risk of cardiovascular mortality increased gradually below an eGFR of approximately 70 ml/min/1.73m^2^ (Fig 2, S3 and S4 Figs).

Of all deaths, 309 deaths (35%) were due to cancer. We found a weak inverse relation between mortality from cancer and kidney function. Due to the low number of deaths from cancer (n = 5) in the lowest category of kidney function, adjusted hazard ratios could not be calculated.

**Fig 3:**
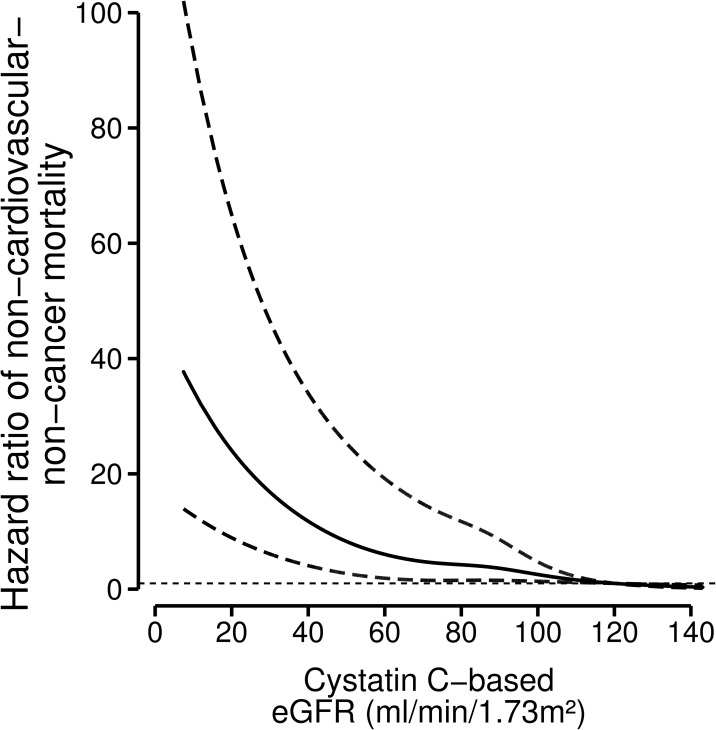
Relation between baseline kidney function and non-cardiovascular-non-cancer mortality with 95%-confidence intervals (dotted lines) among 4561 post-myocardial infarction patients. Hazard ratios for non-cardiovascular-non-cancer causes of mortality depending on kidney function were modeled by separate restricted cubic splines for cystatin C-based kidney function in a Cox-regression model. The model was adjusted for n-3 fatty acids treatment, age, sex, diabetes, current smoking, ratio serum cholesterol-HDL, statin-use, use of anti-hypertensive medication, systolic and diastolic blood pressure. An eGFR of 120 ml/min/1.73m^2^ was taken as the reference point (hazard ratio 1).

Of all deaths, 194 (22%) had non-cardiovascular-non-cancer causes. We found a strong inverse relation between lower kidney function and non-cardiovascular-non-cancer death (Table 2, Fig 3, S5 and S6 Figs). We carried out several sensitivity analyses to compare the effect of eGFR <60 (CKD), and 60–89 with the reference: ≥90 ml/min/1.73m^2^. After exclusion of patients who had cardiovascular disease as secondary cause of death, compared with the reference the hazard ratio for eGFR_cysC_ <60 ml/min/1.73m^2^ for other causes of death was 3.64 (95%-CI: 1.38 to 9.59). Of all non-cardiovascular-non-cancer deaths, 72 were due to diseases of the respiratory system. We found a similar hazard ratio for eGFR_cysC_ <60 ml/min/1.73m^2^ and death due to diseases of the respiratory system of 3.46 (95%-CI: 1.48 to 8.12). Among never smokers the hazard ratio for eGFR_cysC_ <60 ml/min/1.73m^2^ for other causes of death was 3.43 (95%-CI: 0.91–13.0).

## Discussion

This is the largest cohort study of older state-of-the-art drug-treated post-myocardial infarction patients, without severe heart failure, that shows a strong inverse graded relation between kidney function and all-cause mortality. Relative risk of mortality increased linearly below a kidney function of about 80 ml/min/1.73m^2^. Compared to a normal kidney function (≥90 ml/min/1.73m^2^) the multivariable adjusted relative risk for all-cause-mortality was 1.4-fold greater for cystatin C-based kidney function between 60–89 ml/min/1.73m^2^, 2.9-fold for 30–59 ml/min/1.73m^2^ and 4.4-fold for <30 ml/min/1.73m^2^. Impaired kidney function was related to both cardiovascular as well as non-cardiovascular mortality, although its relation with cancer was much weaker.

Our results extend the observations of a meta-analysis that showed a graded inverse relation between kidney function and all-cause mortality.[9] This meta-analysis did not provide relative risks for different age-categories and included patients with and without vascular disease. In a subgroup analysis of prospective studies, including only patients with vascular disease, a 30% lower eGFR was associated with 36% increase in the relative risk of death. We found a similar risk estimate of 37% for all-cause mortality in older cardiac patients comparing an eGFR_cysC_-range of 60–89 with ≥90 ml/min/1.73m^2^. Another meta-analysis, including population-based and high-risk cohorts, showed that individuals aged 65–74 years with an eGFR of 45 or 80 ml/min/1.73m^2^ had a mean all-cause mortality rate of 3.7 and 2.3 per 100 py, respectively.[10] We found lower mortality rates of 2.9 and 1.4 per 100 py in cardiac patients with kidney functions of 30–59 and 60–89 ml/min/1.73m^2^, respectively. However, in contrast to the patients in the Alpha Omega Trial, participants included in this meta-analysis with a history of myocardial infarction enrolled before the year 2000, did most likely not receive optimal cardiovascular drug-treatment such as statins and dual antiplatelet therapy.[10,25,26]

We found that the relative risk of all-cause mortality increased linearly below an eGFR of 80 ml/min/1.73m^2^, which is substantially higher than the threshold of approximately 60 ml/min/1.73m^2^ in the meta-analysis of Hallan *et al*.[10] Most likely, the background risk of all-cause mortality was lower compared to our cohort of post-MI patients because 90% of the individuals were derived from the general population. A large meta-analysis of younger patients (mean age 49 years) without a history of cardiovascular disease showed a similar threshold for creatinine-based kidney function of about 75 ml/min/1.73m^2^ below which the risk for cardiovascular mortality increased steadily.[27] Our results are in line with a large study among post-MI patients with heart failure (>18 years of age), showing that the relative risk of all-cause mortality increased linearly below an eGFR of approximately 75 ml/min/1.73m^2^.[28] In contrast to our study, patients had heart failure, were slightly younger, and not all patients were optimally drug-treated: about 30% used statins and 40% used angiotensin converting enzyme inhibitors.

The present study showed a proportion of cardiovascular and non-cardiovascular mortality in post-MI patients of 42% and 58%, respectively. This is in accordance with findings in the general population, patients with CKD, and (pre-)dialysis patients.[29–32] In our cohort 35% of all patients died from cancer, which was the leading non-cardiovascular cause of death. We found a weak inverse relation between kidney function and death from cancer. Post-MI patients with a kidney function of 30–59 ml/min/1.73m^2^ had a doubled relative risk of death from cancer compared to those with a kidney function ≥90 ml/min/1.73m^2^. Fried *et al*. showed in a community-based cohort of older individuals (mean age 75 years), of whom 21% had a history of cardiovascular disease, a relative risk of 1.30 (0.97 to 1.74) for death from cancer among those with an eGFR_cysC_ <60 compared with an >80 ml/min/1.73m^2^.[33] Growing evidence suggests that aspirin (antiplatelet drug) has a beneficial effect against some types of cancer, particularly of the gastrointestinal tract. Since 84% of all patients of our cohort used aspirin, we could not address this issue. Taken together, in our cohort the relation between kidney function and cancer is much weaker compared with cardiovascular or non-cardiovascular-non-cancer causes of mortality.

We found a strong inverse relation between kidney function and non-cardiovascular-non-cancer mortality. Although we adjusted for smoking, we cannot exclude residual confounding. However, this inverse relation persisted when we confined our analysis to life-time never smokers. After exclusion of patients who had cardiovascular disease as secondary cause of death, the relation between kidney function and other causes of death remained similar. Comparable results were obtained between kidney function and mortality from respiratory diseases. Common traditional cardiovascular risk factors, such as smoking and inflammation, among patients with kidney disease may explain the relation with non-cardiovascular death e.g. respiratory diseases.[34]

Non-GFR determinants of cystatin C, such as inflammation, may enhance the relation between cystatin C-based eGFR and different causes of death.[23] However, additional adjustment for hsCRP in multivariable models only slightly attenuated the relation between kidney function and different causes of death. Moreover, a similar effect attenuation was shown when hsCRP was included in the creatinine-based kidney function models. Most likely, inflammation is an intermediate in the pathway between impaired kidney function and mortality.[35] Therefore, including hsCRP in the model results in over-adjustment and thus, underestimation of the association of impaired kidney function with mortality.

Staging of CKD is used for risk stratification in clinical guidelines to improve patient outcome by identifying high-risk groups. The lower relative mortality risk associated with CKD at older ages has been used as an argument for age-specific CKD diagnosis and staging.[36] However, the present study shows that the CKD-risk relation is also present in older post-myocardial infarction patients. Detection of early stages of CKD may help to improve clinical practice through implementation of targeted therapy to reduce the incidence of adverse events for this large patient group. Renal function does not change over time in one third of the healthy adults, showing the preventability of kidney aging and makes the possibility of retarding kidney function decline through effective treatment plausible.[37]

Our study has several limitations. First, we previously showed in the Alpha Omega Trial that a daily low dose of fish oil retards kidney function decline by 30% in post-myocardial infarction patients.[7] Since patients assigned to fish oil were well-balanced among the four eGFR categories, it is unlikely that trial medication influenced our results. In addition, there was no evidence of effect modification between fish oil treatment and kidney function with regard to mortality (P for interaction = 0.2). Moreover, we adjusted in the present study for the assigned treatment group. Second, GFR was not measured and kidney function was estimated only once at baseline, which may have resulted in nondifferential misclassification of the eGFR categories. However, nondifferential misclassification results generally in effect attenuation.[38] Third, we did not have information on albuminuria or proteinuria, and could therefore not differentiate between patients with or without CKD stage 1 in the reference category. As a result, if anything, we underestimated the strength of the associations of kidney function with different causes of death.

Our prospective cohort study has several strengths. We showed an inverse graded relation between mortality and kidney function based on two different endogenous markers of kidney function, suggesting that impaired kidney function *per se* is a risk factor for mortality. Serum cystatin C and creatinine were analyzed in a central laboratory. Ascertainment of mortality was complete. Of all patients only 6% of kidney function measurements were missing.

We found in older post-myocardial infarction patients who received state-of-the-art drug treatment that kidney function, independent of classic cardiovascular risk factors, predicted all-cause, cardiovascular as well as non-cardiovascular mortality in a graded fashion.

## Supporting information

S1 Table Mortality risks according to four categories of creatinine-based estimated glomerular filtration rate (eGFR). AR absolute risk, CI confidence interval, No number, py person years, Model 1: adjusted for the intervention with n-3 fatty acids, age, sex, diabetes, current smoking, ratio serum cholesterol/HDL, statin-use, anti-hypertensive medication, systolic blood pressure, and diastolic blood pressure. Model 2: in addition to Model 1 additional adjustment for C-reactive protein. eGFR **≥**90 ml/min/1.73m^2^ was taken as the reference category. *Due to the low number of events in the lowest category of eGFR further adjustment could not be performed.
(DOCX)

S2 Table Mortality risks according to four categories of cystatin C-creatinine based estimated glomerular filtration rate (eGFR). AR absolute risk, CI confidence interval, No number, py person years. Model 1: adjusted for the intervention with n-3 fatty acids, age, sex, diabetes, current smoking, ratio serum total cholesterol/HDL, statin-use, anti-hypertensive medication, systolic blood pressure, and diastolic blood pressure. Model 2: in addition to Model 1 additional adjustment for C-reactive protein. eGFR **≥**90 ml/min/1.73m^2^ was taken as the reference category. *Due to the low number of events in the lowest category of eGFR further adjustment could not be performed.
(DOCX)

S1 Fig Relation between baseline creatinine-based kidney function and all-cause mortality with 95%-confidence intervals (dotted lines) among 4561 post-myocardial infarction patients. Hazard ratios for all-cause mortality depending on kidney function were modeled by separate restricted cubic splines for creatinine based-kidney function in a Cox-regression model. The model was adjusted for n-3 fatty acids treatment, age, sex, diabetes, current smoking, ratio serum cholesterol-HDL, statin-use, use of anti-hypertensive medication, systolic and diastolic blood pressure. An eGFR of 120 ml/min/1.73m^2^ was taken as the reference point (hazard ratio 1). The knots were chosen at the 5th, 35th, 65th and 95th percentile of the kidney function distribution, corresponding to creatinine-based eGFR of 43, 77, 94 and 110 ml/min/1.73m^2^.
(TIF)

S2 Fig Relation between baseline combined cystatin C-creatinine kidney function and all-cause mortality with 95%-confidence intervals (dotted lines) among 4561 post-myocardial infarction patients. Hazard ratios for all-cause mortality depending on kidney function were modeled by restricted cubic splines for the combined cystatin C-creatinine based-kidney function in a Cox-regression model. The model was adjusted for n-3 fatty acids treatment, age, sex, diabetes, current smoking, ratio serum cholesterol-HDL, statin-use, use of anti-hypertensive medication, systolic and diastolic blood pressure. An eGFR of 120 ml/min/1.73m^2^ was taken as the reference point (hazard ratio 1). The knots were chosen at the 5th, 35th, 65th and 95th percentile of the kidney function distribution, corresponding to cystatin C-creatinine-based eGFR of 41, 68, 86 and 104 ml/min/1.73m^2^.
(TIF)

S3 Fig Relation between baseline creatinine-based kidney function and cardiovascular mortality with 95%-confidence intervals (dotted lines) among 4561 post-myocardial infarction patients. Hazard ratios for cardiovascular mortality depending on kidney function were modeled by separate restricted cubic splines for creatinine based-kidney function in a Cox-regression model. The model was adjusted for n-3 fatty acids treatment, age, sex, diabetes, current smoking, ratio serum cholesterol-HDL, statin-use, use of anti-hypertensive medication, systolic and diastolic blood pressure. An eGFR of 120 ml/min/1.73m^2^ was taken as the reference point (hazard ratio 1).
(TIF)

S4 Fig Relation between baseline combined cystatin C-creatinine based kidney function and cardiovascular mortality with 95%-confidence intervals (dotted lines) among 4561 post-myocardial infarction patients. Hazard ratios for cardiovascular mortality depending on kidney function were modeled by restricted cubic splines for the combined cystatin C-creatinine based-kidney function in a Cox-regression model. The model was adjusted for n-3 fatty acids treatment, age, sex, diabetes, current smoking, ratio serum cholesterol-HDL, statin-use, use of anti-hypertensive medication, systolic and diastolic blood pressure. An eGFR of 120 ml/min/1.73m^2^ was taken as the reference point (hazard ratio 1).
(TIF)

S5 Fig Relation between baseline creatinine-based kidney function and non-cardiovascular-non-cancer mortality with 95%-confidence intervals (dotted lines) among 4561 post-myocardial infarction patients. Hazard ratios for non-cardiovascular-non-cancer causes of mortality depending on kidney function were modeled by separate restricted cubic splines for creatinine based-kidney function in a Cox-regression model. The model was adjusted for n-3 fatty acids treatment, age, sex, diabetes, current smoking, ratio serum cholesterol-HDL, statin-use, use of anti-hypertensive medication, systolic and diastolic blood pressure. An eGFR of 120 ml/min/1.73m^2^ was taken as the reference point (hazard ratio 1).
(TIF)

S6 Fig Relation between baseline combined cystatin C-creatinine kidney function and non-cardiovascular-non-cancer mortality with 95%-confidence intervals (dotted lines) among 4561 post-myocardial infarction patients. Hazard ratios for non-cardiovascular-non-cancer causes of mortality depending on kidney function were modeled by restricted cubic splines for the combined cystatin C-creatinine based-kidney function in a Cox-regression model. The model was adjusted for n-3 fatty acids treatment, age, sex, diabetes, current smoking, ratio serum cholesterol-HDL, statin-use, use of anti-hypertensive medication, systolic and diastolic blood pressure. An eGFR of 120 ml/min/1.73m^2^ was taken as the reference point (hazard ratio 1).
(TIF)
