# Supplementary material for: Kidney function and specific mortality in 60-80 years old post-myocardial infarction patients: A 10-year follow-up study
Source: PLoS One. 2017 Feb 9;12(2):e0171868. doi: 10.1371/journal.pone.0171868 (PMC5300181; doi:10.1371/journal.pone.0171868)
Supplement: S1 Table — AR absolute risk, CI confidence interval, No number, py person years, Model 1: adjusted for the intervention with n-3 fatty acids, age, sex, diabetes, current smoking, ratio serum cholesterol/HDL, statin-use, anti-hypertensive medication, systolic blood pressure, and diastolic blood pressure. Model 2: in addition to Model 1 additional adjustment for C-reactive protein. eGFR ≥90 ml/min/1.73m2 was taken as the reference category. *Due to the low number of events in the lowest category of eGFR further adjustment could not be performed. (DOCX) [file pone.0171868.s001.docx]

| **Creatinine-based eGFR,** ml/min/1.73m^2^ | **≥90** | **60-89** | **30-59** | **<30** | **P for Trend** |
| --- | --- | --- | --- | --- | --- |
|  |  |  |  |  |  |
| **All-cause mortality** |  |  |  |  |  |
| No patients | 2089 | 1714 | 706 | 52 |  |
| Person-years (py) | 13878.18 | 10689.62 | 4027.04 | 245.57 |  |
| No deaths | 283 | 358 | 207 | 25 |  |
| AR per 100 py (95%-CI) | 2.04 (1.82 to 2.29) | 3.35 (3.02 to 3.71) | 5.14 (4.50 to 5.87) | 10.18 (6.99 to 14.60) |  |
|  |  |  |  |  |  |
| Crude | 1 | 1.68 (1.44 to 1.96) | 2.66 (2.23 to 3.19) | 5.47 (3.63 to 8.23) | <0.001 |
| Age & sex adj. | 1 | 1.28 (1.08 to 1.51) | 1.91 (1.58 to 2.32) | 3.25 (2.14 to 4.94) | <0.001 |
| Model 1 | 1 | 1.25 (1.06 to 1.48) | 1.85 (1.52 to 2.25) | 2.85 (1.87 to 4.35) | <0.001 |
| Model 2 | 1 | 1.24 (1.05 to 1.47) | 1.81 (1.49 to 2.21) | 2.80 (1.83 to 4.26) | <0.001 |
|  |  |  |  |  |  |
| **Cardiovascular mortality** |  |  |  |  |  |
| No deaths | 99 | 162 | 95 | 14 |  |
| Crude | 1 | 2.18 (1.70 to 2.80) | 3.50 (2.64 to 4.64) | 8.70 (4.96 to 15.23) | <0.001 |
| Age & sex adj. | 1 | 1.62 (1.24 to 2.12) | 2.44 (1.81 to 3.30) | 5.02 (2.82 to 8.95) | <0.001 |
| Model 1 | 1 | 1.58 (1.21 to 2.06) | 2.33 (1.72 to 3.16) | 4.35 (2.43 to 7.78) | <0.001 |
| Model 2 | 1 | 1.56 (1.19 to 2.04) | 2.29 (1.69 to 3.12) | 4.27 (2.39 to 7.64) | <0.001 |
|  |  |  |  |  |  |
| **Cancer mortality** |  |  |  |  |  |
| No deaths | 117 | 120 | 69 | 3 |  |
| Crude | 1 | 1.35 (1.05 to 1.75) | 2.13 (1.58 to 2.87) | 1.59 (0.50 to 5.00) | <0.001 |
| Age & sex adj. | 1 | 1.06 (0.81 to 1.39) | 1.59 (1.16 to 2.19) | * |  |
| Model 1 | 1 | 1.06 (0.80 to 1.39) | 1.60 (1.16 to 2.20) | * |  |
| Model 2 | 1 | 1.05 (0.80 to 1.38) | 1.56 (1.13 to 2.16) | * |  |
|  |  |  |  |  |  |
| **Non-cardiovascular/ non-cancer mortality** |  |  |  |  |  |
| No deaths | 67 | 76 | 43 | 8 |  |
| Crude | 1 | 1.51 (1.09 to 2.10) | 2.37 (1.61 to 3.47) | 7.48 (3.59 to 15.59) | <0.001 |
| Age & sex adj. | 1 | 1.15 (0.81 to 1.64) | 1.68 (1.11 to 2.54) | 4.18 (1.96 to 8.92) | <0.001 |
| Model 1 | 1 | 1.11 (0.77 to 1.58) | 1.56 (1.02 to 2.39) | 3.44 (1.60 to 7.40) | 0.004 |
| Model 2 | 1 | 1.09 (0.76 to 1.56) | 1.53 (1.00 to 2.34) | 3.38 (1.57 to 7.26) | 0.005 |
