# Supplementary material for: Kidney function and specific mortality in 60-80 years old post-myocardial infarction patients: A 10-year follow-up study
Source: PLoS One. 2017 Feb 9;12(2):e0171868. doi: 10.1371/journal.pone.0171868 (PMC5300181; doi:10.1371/journal.pone.0171868)
Supplement: S2 Table — AR absolute risk, CI confidence interval, No number, py person years. Model 1: adjusted for the intervention with n-3 fatty acids, age, sex, diabetes, current smoking, ratio serum total cholesterol/HDL, statin-use, anti-hypertensive medication, systolic blood pressure, and diastolic blood pressure. Model 2: in addition to Model 1 additional adjustment for C-reactive protein. eGFR ≥90 ml/min/1.73m2 was taken as the reference category. *Due to the low number of events in the lowest category of eGFR further adjustment could not be performed. (DOCX) [file pone.0171868.s002.docx]

| **CysC-creat-based eGFR,** ml/min/1.73m^2^ | **≥90** | **60-89** | **30-59** | **<30** | **P for Trend** |
| --- | --- | --- | --- | --- | --- |
|  |  |  |  |  |  |
| **All-cause mortality** |  |  |  |  |  |
| No patients | 1236 | 2298 | 944 | 83 |  |
| Person-years (py) | 8359.82 | 14711.15 | 5363.84 | 405.59 |  |
| No deaths | 128 | 393 | 313 | 39 |  |
| AR per 100 py (95%-CI) | 1.53 (1.33 to 1.77) | 2.67 (2.42 to 2.94) | 5.84 (5.24 to 6.50) | 9.62 (7.11 to 12.88) |  |
|  |  |  |  |  |  |
| Crude | 1 | 1.78 (1.46 to 2.17) | 4.03 (3.28 to 4.96) | 6.90 (4.82 to 9.89) | <0.001 |
| Age & sex adj. | 1 | 1.41 (1.15 to 1.74) | 2.67 (2·14 to 3.34) | 4.29 (2.96 to 6.23) | <0.001 |
| Model 1 | 1 | 1.35 (1.10 to 1.66) | 2.40 (1.91 to 3.01) | 3.91 (2.68 to 5.71) | <0.001 |
| Model 2 | 1 | 1.33 (1.08 to 1.63) | 2.32 (1.85 to 2.91) | 3.74 (2.56 to 5.45) | <0.001 |
|  |  |  |  |  |  |
| **Cardiovascular mortality** |  |  |  |  |  |
| No deaths | 45 | 157 | 148 | 20 |  |
| Crude | 1 | 2.02 (1.45 to 2.82) | 5.43 (3.88 to 7.58) | 10.02 (5.91 to 16.98) | <0.001 |
| Age & sex adj. | 1 | 1.64 (1.16 to 2.30) | 3.70 (2.58 to 5.31) | 6.43 (3.71 to 11.14) | <0.001 |
| Model 1 | 1 | 1.60 (1.14 to 2.26) | 3.36 (2.33 to 4.85) | 5.98 (3.43 to 10.45) | <0.001 |
| Model 2 | 1 | 1.58 (1.12 to 2.23) | 3.27 (2.26 to 4.72) | 5.75 (3.29 to 10.06) | <0.001 |
|  |  |  |  |  |  |
| **Cancer mortality** |  |  |  |  |  |
| No deaths | 61 | 150 | 93 | 5 |  |
| Crude | 1 | 1.42 (1.06 to 1.91) | 2.50 (1.81 to 3.45) | 1.85 (0.74 to 4.61) | <0.001 |
| Age & sex adj. | 1 | 1.21 (0.89 to 1.65) | 1.86 (1.31 to 2.65) | * |  |
| Model 1 | 1 | 1.16 (0.85 to 1.58) | 1.75 (1.22 to 2.50) | * |  |
| Model 2 | 1 | 1.13 (0.83 to 1.54) | 1.68 (1.17 to 2.41) | * |  |
|  |  |  |  |  |  |
| **Non-cardiovascular/ non-cancer mortality** |  |  |  |  |  |
| No deaths | 22 | 86 | 72 | 14 |  |
| Crude | 1 | 2.27 (1.42 to 3.63) | 5.47 (3.39 to 8.82) | 14.67 (7.50 to 28.72) | <0.001 |
| Age & sex adj. | 1 | 1.55 (0.95 to 2.51) | 2.80 (1.67 to 4.69) | 6.89 (3.41 to 13.93) | <0.001 |
| Model 1 | 1 | 1.41 (0.87 to 2.30) | 2.29 (1.35 to 3.88) | 5.54 (2.71 to 11.33) | <0.001 |
| Model 2 | 1 | 1.39 (0.85 to 2.26) | 2.22 (1.31 to 3.76) | 5.28 (2.57 to 10.83) | <0.001 |
