# Supplementary figures and images for: Kidney function and specific mortality in 60-80 years old post-myocardial infarction patients: A 10-year follow-up study
Source: PLoS One. 2017 Feb 9;12(2):e0171868. doi: 10.1371/journal.pone.0171868 (PMC5300181; doi:10.1371/journal.pone.0171868)

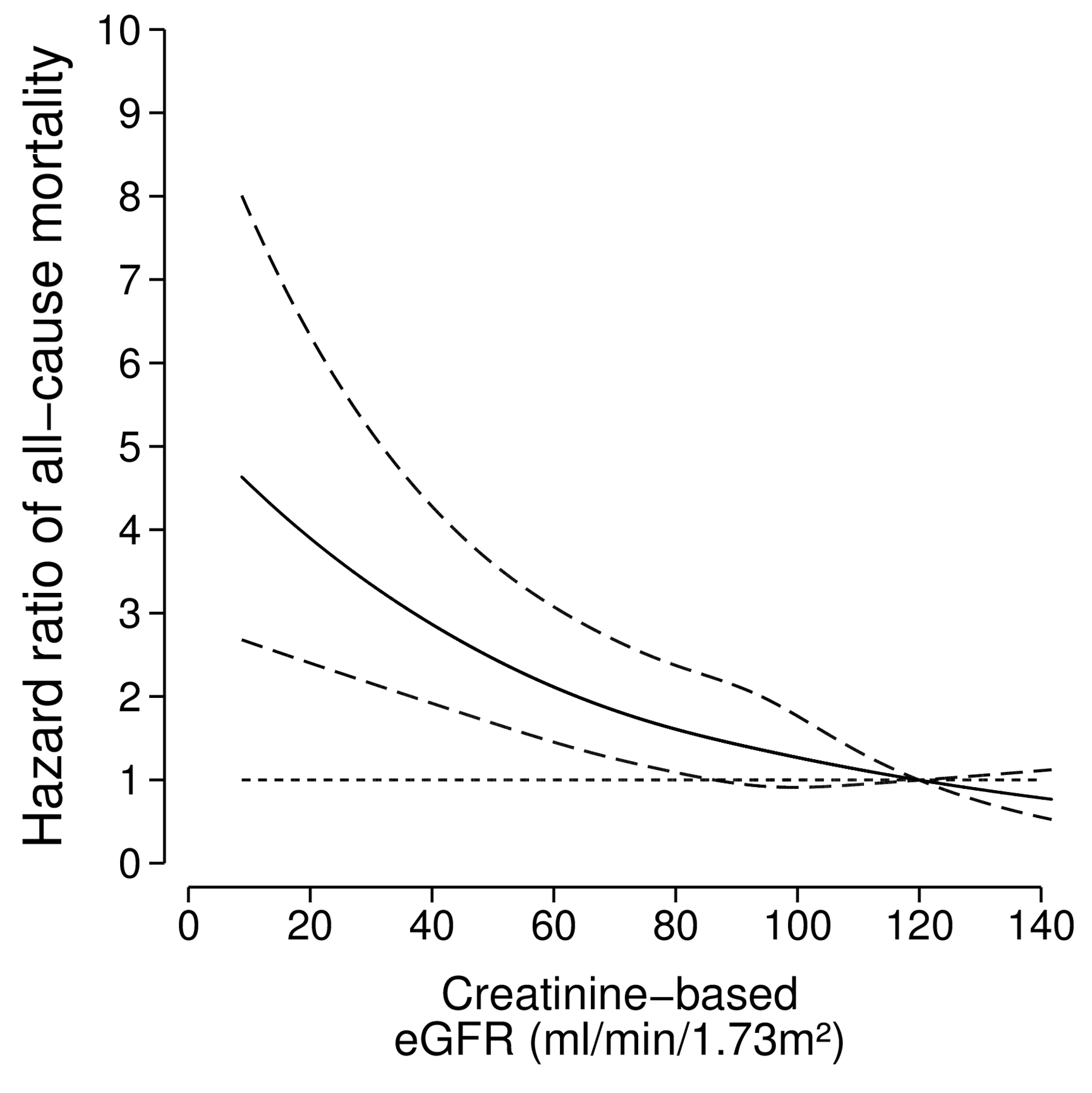

Supplement: S1 Fig — Hazard ratios for all-cause mortality depending on kidney function were modeled by separate restricted cubic splines for creatinine based-kidney function in a Cox-regression model. The model was adjusted for n-3 fatty acids treatment, age, sex, diabetes, current smoking, ratio serum cholesterol-HDL, statin-use, use of anti-hypertensive medication, systolic and diastolic blood pressure. An eGFR of 120 ml/min/1.73m2 was taken as the reference point (hazard ratio 1). The knots were chosen at the 5th, 35th, 65th and 95th percentile of the kidney function distribution, corresponding to creatinine-based eGFR of 43, 77, 94 and 110 ml/min/1.73m2. (TIF) [file pone.0171868.s003.tif]

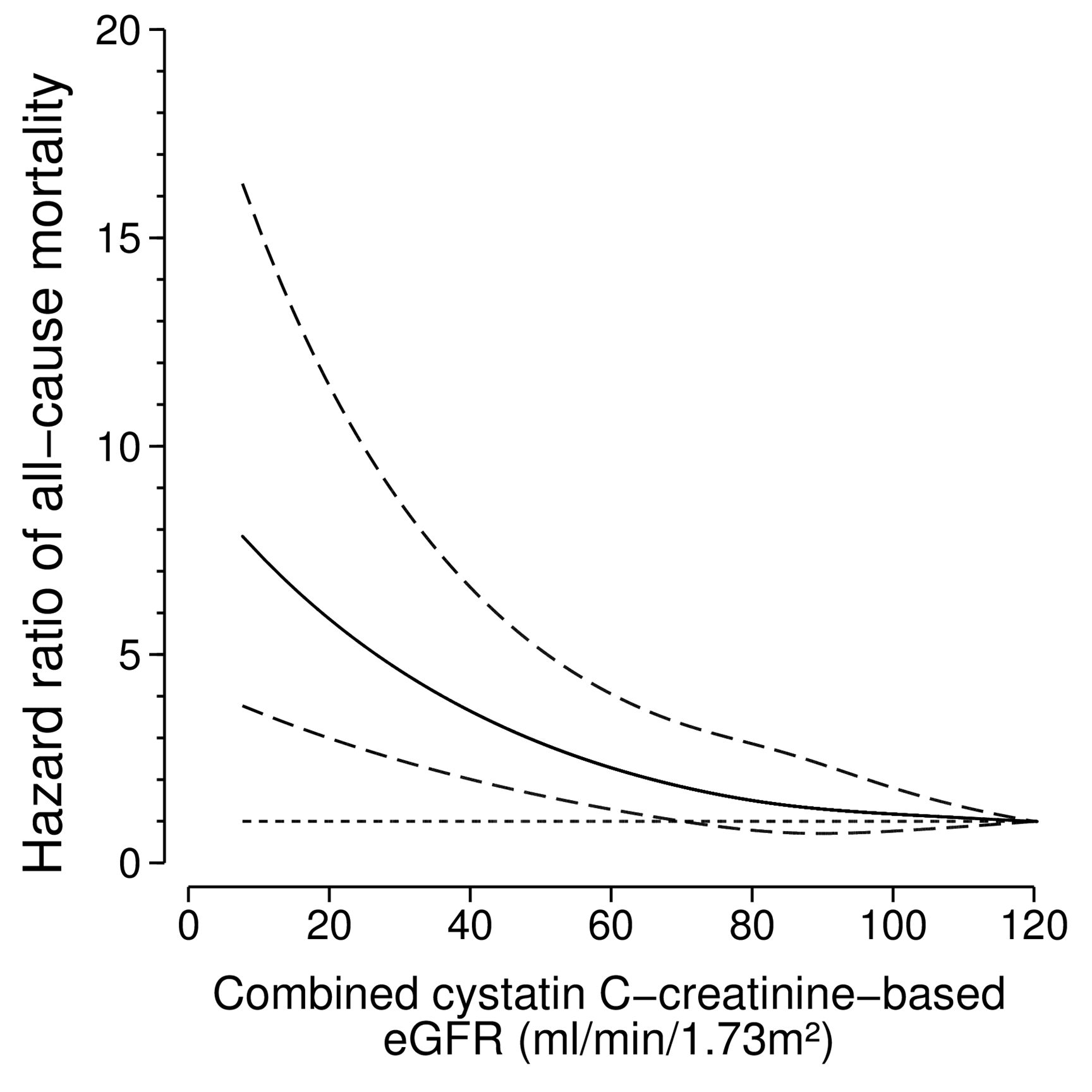

Supplement: S2 Fig — Hazard ratios for all-cause mortality depending on kidney function were modeled by restricted cubic splines for the combined cystatin C-creatinine based-kidney function in a Cox-regression model. The model was adjusted for n-3 fatty acids treatment, age, sex, diabetes, current smoking, ratio serum cholesterol-HDL, statin-use, use of anti-hypertensive medication, systolic and diastolic blood pressure. An eGFR of 120 ml/min/1.73m2 was taken as the reference point (hazard ratio 1). The knots were chosen at the 5th, 35th, 65th and 95th percentile of the kidney function distribution, corresponding to cystatin C-creatinine-based eGFR of 41, 68, 86 and 104 ml/min/1.73m2. (TIF) [file pone.0171868.s004.tif]

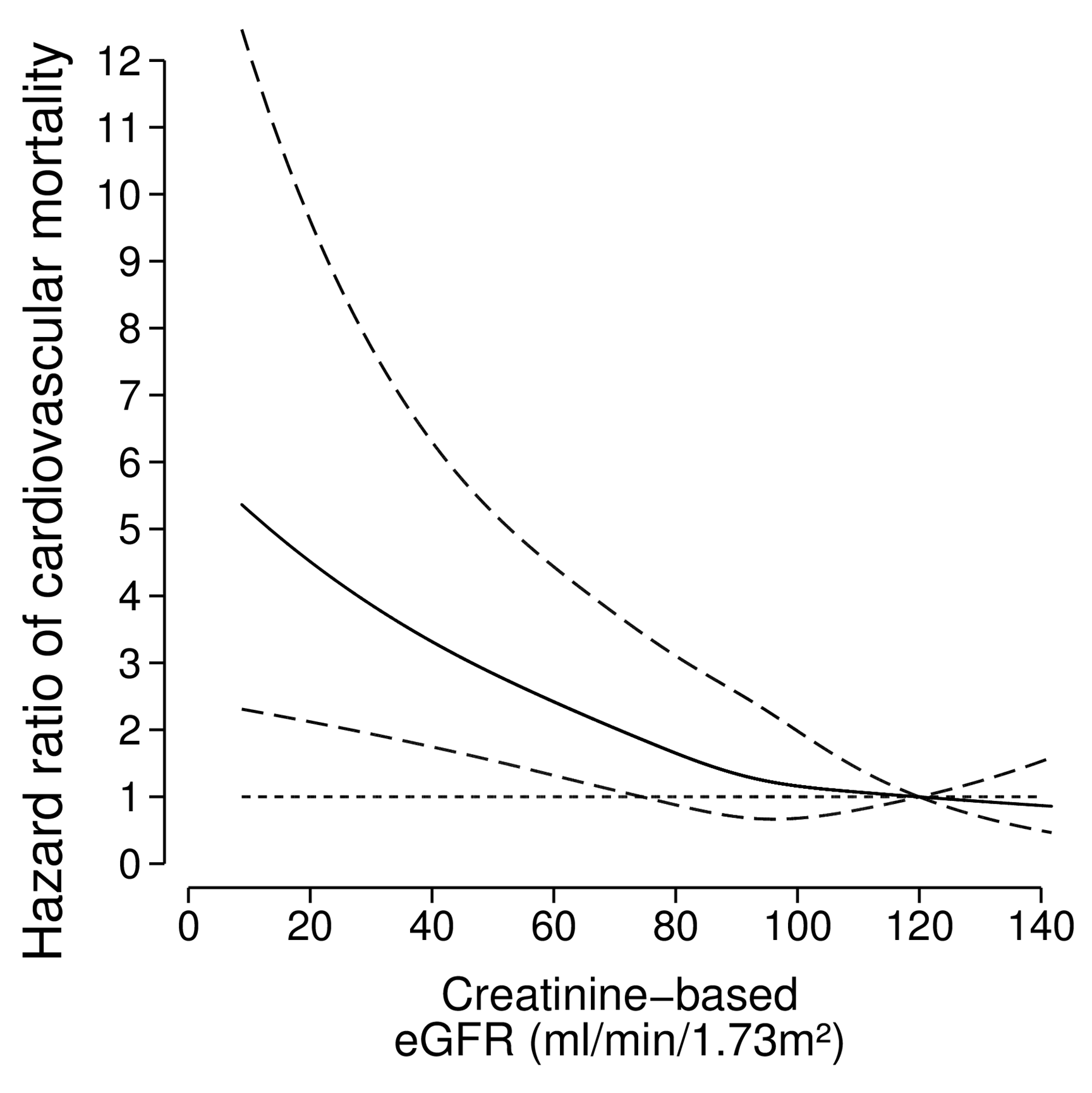

Supplement: S3 Fig — Hazard ratios for cardiovascular mortality depending on kidney function were modeled by separate restricted cubic splines for creatinine based-kidney function in a Cox-regression model. The model was adjusted for n-3 fatty acids treatment, age, sex, diabetes, current smoking, ratio serum cholesterol-HDL, statin-use, use of anti-hypertensive medication, systolic and diastolic blood pressure. An eGFR of 120 ml/min/1.73m2 was taken as the reference point (hazard ratio 1). (TIF) [file pone.0171868.s005.tif]

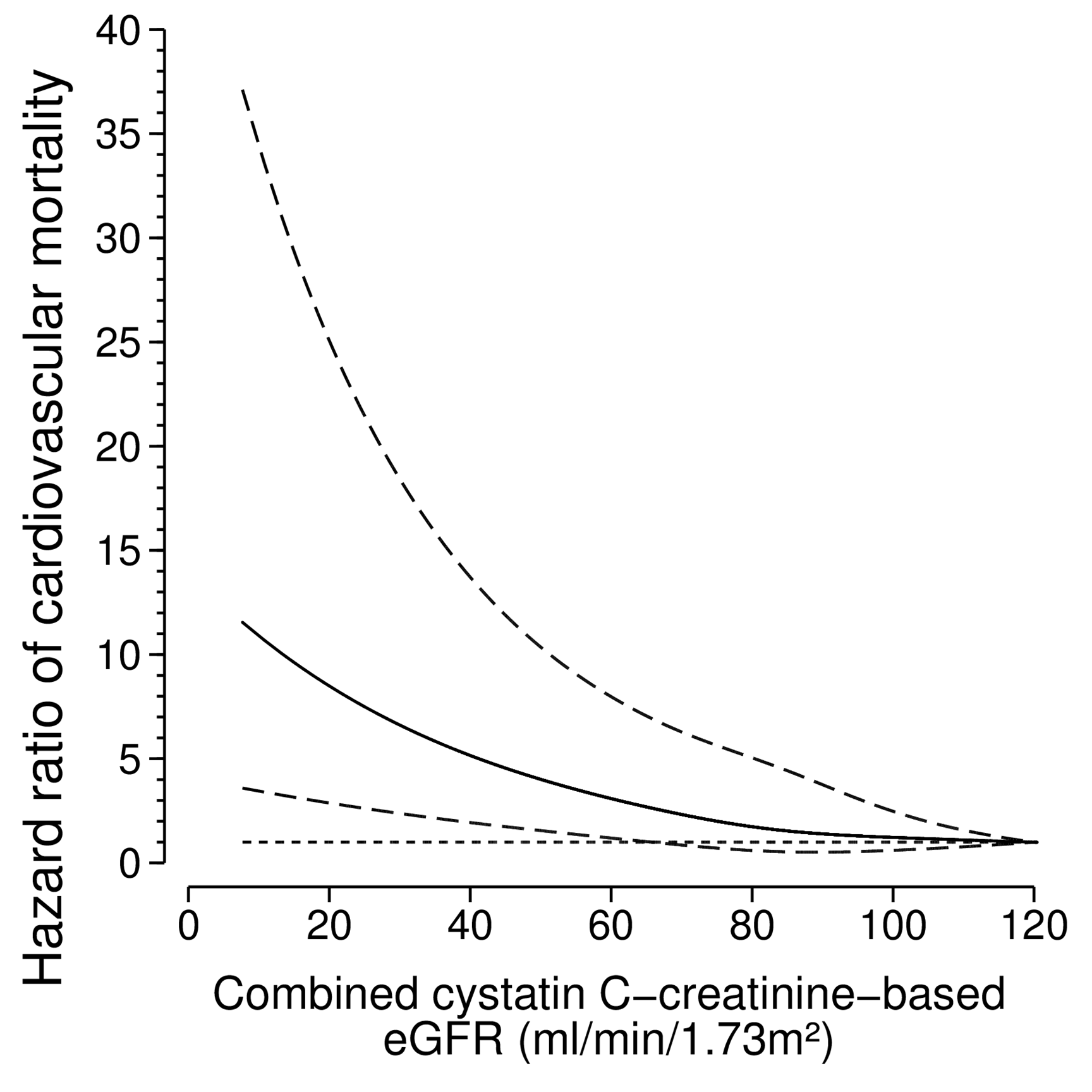

Supplement: S4 Fig — Hazard ratios for cardiovascular mortality depending on kidney function were modeled by restricted cubic splines for the combined cystatin C-creatinine based-kidney function in a Cox-regression model. The model was adjusted for n-3 fatty acids treatment, age, sex, diabetes, current smoking, ratio serum cholesterol-HDL, statin-use, use of anti-hypertensive medication, systolic and diastolic blood pressure. An eGFR of 120 ml/min/1.73m2 was taken as the reference point (hazard ratio 1). (TIF) [file pone.0171868.s006.tif]

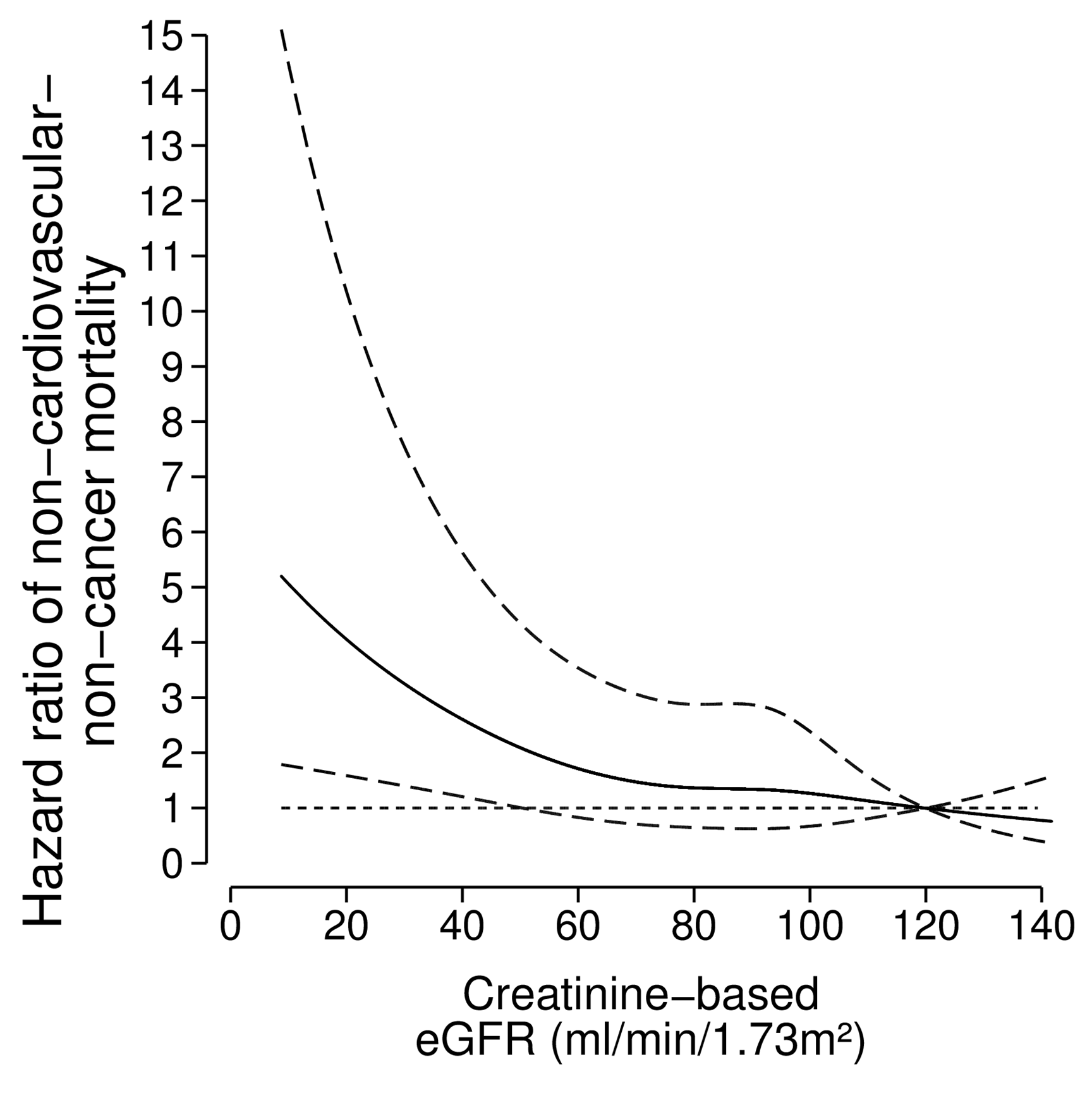

Supplement: S5 Fig — Hazard ratios for non-cardiovascular-non-cancer causes of mortality depending on kidney function were modeled by separate restricted cubic splines for creatinine based-kidney function in a Cox-regression model. The model was adjusted for n-3 fatty acids treatment, age, sex, diabetes, current smoking, ratio serum cholesterol-HDL, statin-use, use of anti-hypertensive medication, systolic and diastolic blood pressure. An eGFR of 120 ml/min/1.73m2 was taken as the reference point (hazard ratio 1). (TIF) [file pone.0171868.s007.tif]

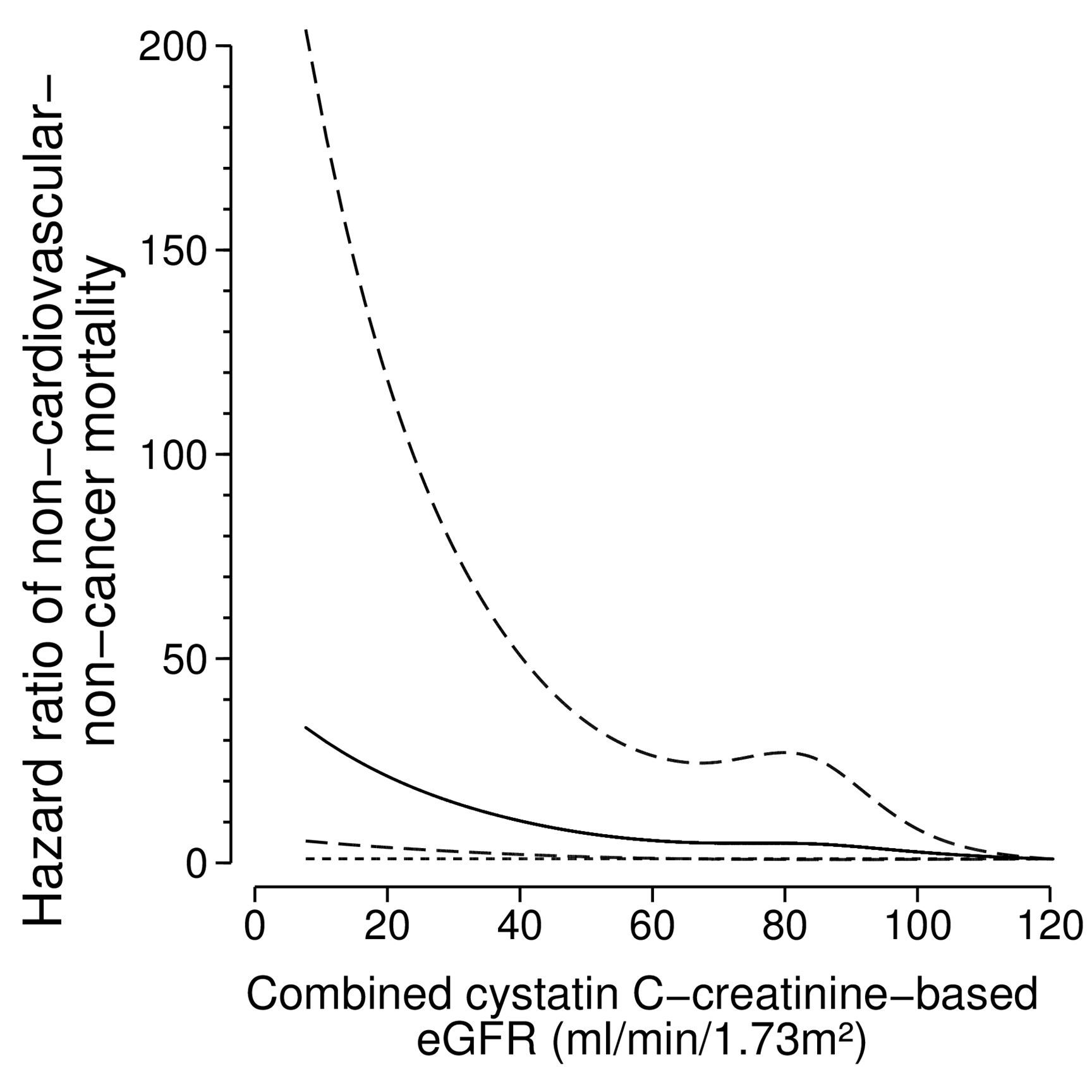

Supplement: S6 Fig — Hazard ratios for non-cardiovascular-non-cancer causes of mortality depending on kidney function were modeled by restricted cubic splines for the combined cystatin C-creatinine based-kidney function in a Cox-regression model. The model was adjusted for n-3 fatty acids treatment, age, sex, diabetes, current smoking, ratio serum cholesterol-HDL, statin-use, use of anti-hypertensive medication, systolic and diastolic blood pressure. An eGFR of 120 ml/min/1.73m2 was taken as the reference point (hazard ratio 1). (TIF) [file pone.0171868.s008.tif]
